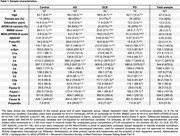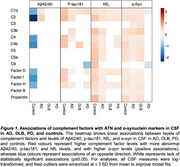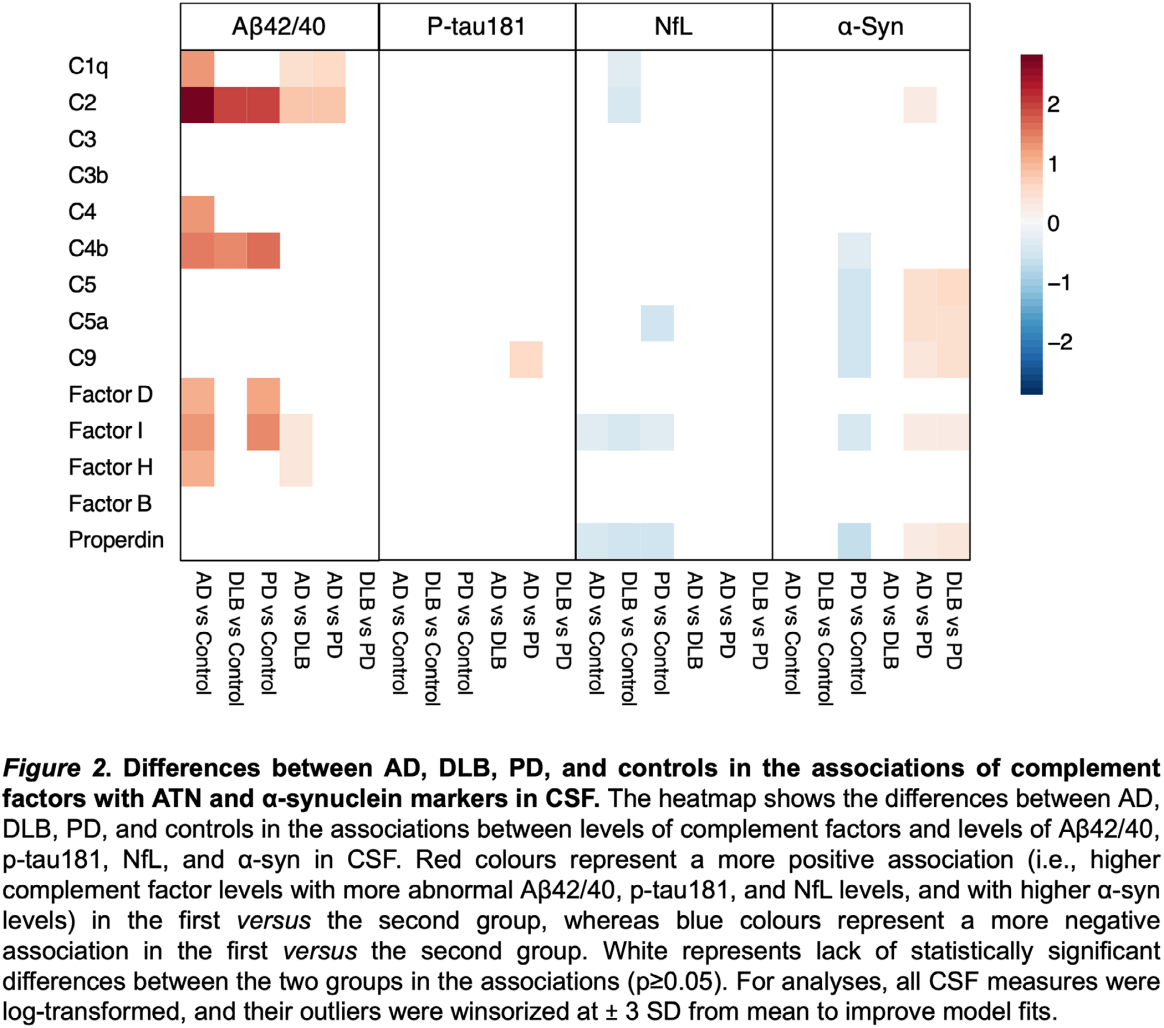# Associations between CSF complement factors and biomarkers of amyloid, tau, neurofilament light chain, and α‐synuclein in AD, DLB, and PD

**DOI:** 10.1002/alz70856_104363

**Published:** 2025-12-26

**Authors:** Marianna Rizzo, Charlotte E. Teunissen, Frederic Brosseron, Sandra Kuhs, Gwendlyn Kollmorgen, Henrik Zetterberg, Kaj Blennow, Rejko Krüger, Sara B. Gomes Fernandes, Dag Aarsland, Olga Borejko, Davit Chokoshvili, Wiesje M. van der Flier, Afina W. Lemstra, Betty M. Tijms, Gabor C Petzold, Annika Spottke, Giovanni B. Frisoni, Kathrin Brockmann, Thomas Gasser, Tormod Fladby, Marianne Wettergreen, Frank Jessen, Emrah Düzel, Günter U Höglinger, Claire Chevalier, Rajaraman Krishnan, Pieter Jelle Visser, Stephanie J. B. Vos, the IMI EPND consortium

**Affiliations:** ^1^ Alzheimer Center Limburg, School for Mental Health and Neuroscience, Maastricht University, Maastricht, Netherlands; ^2^ Neurochemistry Laboratory, Department of Laboratory Medicine, Amsterdam Neuroscience, Amsterdam UMC, Vrije Universiteit Amsterdam, Amsterdam, Netherlands; ^3^ German Center for Neurodegenerative Diseases (DZNE), Venusberg‐Campus 1, 53127, Bonn, Germany; ^4^ Roche Diagnostics GmbH, Penzberg, Germany; ^5^ Department of Neurodegenerative Disease, UCL Institute of Neurology, London, United Kingdom; ^6^ Wisconsin Alzheimer's Disease Research Center, University of Wisconsin School of Medicine and Public Health, Madison, WI, USA; ^7^ Department of Psychiatry and Neurochemistry, Institute of Neuroscience and Physiology, The Sahlgrenska Academy, University of Gothenburg, Mölndal, Gothenburg, Sweden; ^8^ Clinical Neurochemistry Laboratory, Sahlgrenska University Hospital, Mölndal, Västra Götaland län, Sweden; ^9^ Department of Psychiatry and Neurochemistry, Institute of Neuroscience and Physiology, The Sahlgrenska Academy, University of Gothenburg, Mölndal, Sweden; ^10^ Clinical Neurochemistry Laboratory, Sahlgrenska University Hospital, Mölndal, Sweden; ^11^ Transversal Translational Medicine, Luxembourg Institute of Health, Strassen, Luxembourg; ^12^ Luxembourg Centre for Systems Biomedicine, University of Luxembourg, Esch‐sur‐Alzette, Luxembourg; ^13^ Parkinson Research Clinic, Centre Hospitalier de Luxembourg, Luxembourg, Luxembourg; ^14^ Institute of Psychiatry, Psychology and Neuroscience, King's College London, London, United Kingdom; ^15^ Centre for Age‐Related Medicine, Stavanger University Hospital, Stavanger, Stavanger, Norway; ^16^ Luxembourg National Data Service, Esch‐sur‐Alzette, Luxembourg; ^17^ Alzheimer Center Amsterdam, Department of Neurology, Amsterdam Neuroscience, Vrije Universiteit Amsterdam, Amsterdam UMC, Amsterdam, Netherlands; ^18^ Department of Epidemiology and Data Science, Vrije Universiteit Amsterdam, Amsterdam UMC, Amsterdam, North Holland, Netherlands; ^19^ German Center for Neurodegenerative Diseases (DZNE), Bonn, NRW, Germany; ^20^ Division of Vascular Neurology, Department of Neurology, University Hospital Bonn, Bonn, NRW, Germany; ^21^ Department of Neurology, University of Bonn, Bonn, Germany; ^22^ Memory Clinic, Geneva University Hospitals, Geneva, Switzerland; ^23^ Laboratory of Neuroimaging of Aging, University of Geneva, Geneva, Switzerland; ^24^ Hertie Institute for Clinical Brain Research, Department of Neurodegenerative Diseases, University of Tübingen, Tübingen, Germany; ^25^ German Center for Neurodegenerative Diseases (DZNE), Tübingen, Germany; ^26^ Department of Neurology, Akershus University Hospital, Lørenskog, Norway; ^27^ Institute of Clinical Medicine, University of Oslo, Oslo, Norway; ^28^ Department of Psychiatry, University of Cologne, Medical Faculty, Kerpener Strasse 62, Cologne, Germany; ^29^ Excellence Cluster on Cellular Stress Responses in Aging‐Associated Diseases (CECAD), University of Cologne, Cologne, Germany; ^30^ German Center for Neurodegenerative Diseases (DZNE), Bonn, Germany; ^31^ German Center for Neurodegenerative Diseases (DZNE), Magdeburg, Germany; ^32^ Institute of Cognitive Neurology and Dementia Research (IKND), Otto‐von‐Guericke University, Magdeburg, Sachsen Anhalt, Germany; ^33^ German Center for Neurodegenerative Diseases (DZNE), Feodor‐Lynen‐Strasse 17, 81377, Munich, Germany; ^34^ Department of Neurology, University Hospital, LMU Munich, Munich, Bavaria, Germany; ^35^ Sanofi, Cambridge, MA, USA; ^36^ Department of Neurobiology, Care Sciences and Society, Division of Neurogeriatrics, Karolinska Institutet, Stockholm, Sweden

## Abstract

**Background:**

While evidence suggests complement system involvement in Alzheimer's disease (AD), dementia with Lewy bodies (DLB), and Parkinson's disease (PD), its association with disease biomarkers remains unclear. We investigated the relationship of complement factors with amyloid, tau, NfL, and α‐synuclein in CSF in AD, DLB, PD, and controls.

**Method:**

We included 321 individuals with AD, DLB, PD, and controls from 6 centers of the EPND study. CSF Aβ42/40, *p*‐tau181, NfL, and α‐syn were centrally measured using NeuroToolKit (Roche Diagnostics), and 14 CSF complement factors using Milliplex (Merck KGaA). Controls were defined as normal cognition and normal Aβ42/40, whereas AD as abnormal Aβ42/40 without meeting clinical criteria of DLB or PD. Linear regression models adjusted for age and sex were used. Associations were post‐hoc compared between individuals with low(≤23), intermediate(24‐27), and high(≥28) MMSE scores.

**Result:**

Sample characteristics are presented in Table 1. Lower Aβ42/40 levels were associated with lower levels of 7 complement factors in controls and with higher C1q and C2 levels specifically in AD (Figure 1, Figure 2). No associations of Aβ42/40 with complement were found in DLB and PD. Higher *p*‐tau181 levels were associated with increased levels of 7 complement factors in controls and 6 in AD, and showed fewer associations in DLB and PD. The strength of *p*‐tau181 associations with complement was similar across groups. Higher NfL levels were widely associated with higher complement factor levels in controls (13) and AD (12), and less in PD (6) and DLB (4). Higher α‐syn levels were broadly associated with higher complement factor levels in AD (13), controls (12), and DLB (12), but only minimally in PD (1). The strength of these NfL and α‐syn associations with complement was not disease‐specific. Conversely, compared to all groups, in PD higher α‐syn levels were associated with lower C5, C5a, C9, factor‐I and properdin levels. Individuals with intermediate MMSE scores largely drove the associations of α‐syn with complement in AD. MMSE level did not clearly impact other associations.

**Conclusion:**

CSF complement factors were associated with amyloid, tau, NfL, and α‐synuclein, suggesting complement system involvement in several neurodegenerative diseases. Complement showed disease‐specific associations with amyloid in AD and α‐synuclein in PD.